# Life lessons

**DOI:** 10.3762/bjoc.11.256

**Published:** 2015-11-27

**Authors:** Jonathan R Nitschke

**Affiliations:** 1 Department of Chemistry, University of Cambridge, Lensfield Road, Cambridge, CB2 1EW, United Kingdom

**Keywords:** autobiography, host–guest, self-assembly, supramolecular

## Abstract

*Reminiscing about his younger self:* “I mean I can’t very well just 86 [in American slang, to “86” is to eject, remove, or discard someone or something, J.R.N.] this guy from my life. On the other hand, if through some as yet undeveloped technology I were to run into him today, how comfortable would I feel about lending him money, or for that matter even stepping down the street to have a beer and talk over old times?” ― Thomas Pynchon, *Slow Learner*

## Review

I was raised in Syracuse, New York (USA), and went to a series of state schools of varying quality. Only the faces of the bad teachers stick with me, but I remember the names, too, of a few of the good ones – Karen Curry (High School Biology), Michelle Grosnick (Earth Science). Following my parents’ divorce I moved to Gainesville, Florida when I was 16, where I enrolled in the International Baccalaureate program of Eastside High School. I had been drawn to chemistry for a few years by then. My chemistry teacher, Susan Zoltewicz (wife of Professor John Zoltewicsz, University of Florida), recognized my interest, and very kindly arranged for me to do a weekly afterschool apprenticeship with a lab technician named Charlie, whom I helped to set up all manner of chemical demonstrations and experiments at the University of Florida.

I went on to study at Williams College in Massachusetts. I knew chemistry was going to be my major subject, but my interests were broad, leading me to think that a liberal arts education might be a good fit. I remember greatly enjoying courses on art history and 20^th^ century German history, along with a tutorial-style course on heterocyclic chemistry by J. Hodge Markgraf. Two summers stand out in my memory, when I carried out internships at Nanoptics, a company in Gainesville that makes optical fiber and various devices that incorporate it. The company was small enough at the time for me to enjoy considerable interaction with its founder and CEO, Jim Walker, who had been in charge of internally-initiated physics experiments at Fermilab. I had several challenging and very engaging jobs to do, including putting a disassembled fiber-spinning machine back together, and programming a microcontroller to run a stepper motor. I’m pretty sure that Jim’s good word got me in to Berkeley for Ph.D. studies, despite mediocre results for my senior project (with Lee Park at Williams) and during a summer internship at SRI in Menlo Park, California.

I arrived at Berkeley with the impression of having gotten in by the skin of my teeth – that I would have to work twice as hard as anyone else just to scrape through. The first year of the program was a trial by fire, involving coursework – an excellent Physical Organic Chemistry course taught by Bob Bergman stood out – teaching a laboratory section, choosing a supervisor, and racing to get work done for the crucial first-year report. I remember waking up at 4 am during my first year, deeply concerned that I couldn’t get a reaction to work consistently, and so heading in to lab to have another go. Although this was an incredibly poor idea from a safety perspective, the underlying attitude served me well. I loved the environment at Berkeley – the place was fizzing with intellectual energy, manifested both as world-class scholarship and as Hunter S. Thompson-style craziness.

**Scheme 1:**
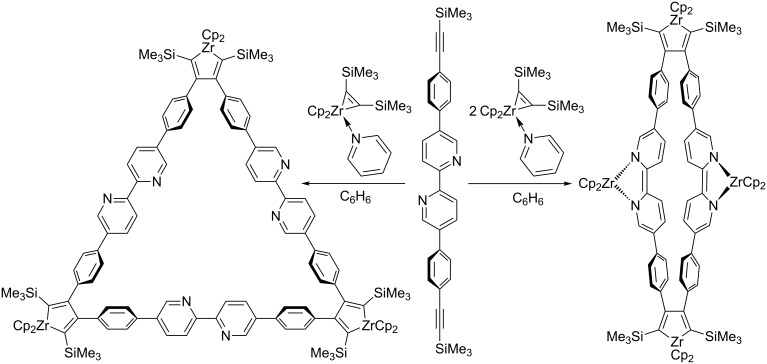
Zirconocene-coupled dimeric (right) and trimeric (left) macrocycles [1–2].

Under T. Don Tilley’s supervision, my Ph.D. work involved the development of zirconocene-mediated macrocyclization reactions to make a series of new structures under thermodynamic control, two examples of which are shown in Scheme 1 [1–2].

This Ph.D. work gave me a taste for organic chemistry involving transition metals, and an interest in structures that form under conditions of thermodynamic equilibration.

Towards the end of my Ph.D. work, Jean-Marie Lehn gave a talk at Berkeley, a highlight of which was some of Bernie Hasenknopf’s latest work on circular helicates [3–4]. I remember being greatly impressed by the intricacy and beauty of these assemblies, and struck by the relative simplicity of their precursors. I spent considerable time over the following weeks digging through the literature, which involved quite a bit more physical activity in those pre-digitization days, in pulling great volumes down from high shelves, reading the work, photocopying the most interesting papers, and tracing back through the references for other papers, to gain context and depth. I came away convinced that was the kind of chemistry that I wanted to do for a postdoc.

So I wrote to Jean-Marie, and ultimately was offered a place in his labs in Strasbourg. I asked two Berkeley alumni who had recently finished postdocs there about their experiences, and was advised quite strongly against going! It’s too difficult to get work done there, they said, and the French don’t like Americans. A bit of back-and-forth led me to conclude that I might make it through OK if I polished up my high-school French, and tried to tone down some of the American cultural characteristics that seemed least compatible with the French worldview – the ‘in your face’ attitude that can sometimes compel action in the US, I reckoned, would likely backfire in France.

After a slow start, I ended up getting some good results in a project related to some new dynamic-covalent grid complexes [5]. This work didn’t come to fruition before I went onto the academic job market, however! I thus had few US interviews, and only one offer from Case Western by the end of my time at Strasbourg. I had also applied for a ‘maître-assistant’ position at the University of Geneva in Switzerland, thinking of it mostly as practice for the ‘real’ US interviews. The interview went well; I was promised full scientific independence and the opportunity to supervise two Ph.D. students. In the end I chose Geneva because everything I needed was there, the teaching load was relatively light, and it seemed that I might stand a better chance of recruiting good students there. The senior members of the Department and School also impressed me; it seemed that advice and mentoring would be available for the asking. These first impressions held up well with time.

The group’s first two Ph.D. students were hired from the University of Strasbourg, where there was no shortage of very well qualified M.Sc. students in search of opportunity. I interviewed several and extended offers to the two best, David Schultz and Marie Hutin, who did not let me down. Their intelligence and hard work laid the foundations of the group’s work to the present day. Just after concluding the interviews, I was called to Berne, where a senior chemistry professor interviewed me on the Swiss National Science Foundation proposal that I had submitted. I did not make out so well as Marie and David, being informed that the work that I proposed was much too ambitious – it would require the mastery of techniques and concepts to which I had never been exposed. “Write a new proposal,” came the advice, “convince us that you can get good work done quickly with limited means.”

Although this rejection was devastating, time has told that it was the best advice that I could have gotten at that point in my career, delivered in a context that I could not ignore. My senior colleagues at Geneva came through with bridge funding so that I could still give Marie and David their promised places – a kindness for which I remain grateful – and the next proposal I wrote obtained modest funding.

**Figure 1:**
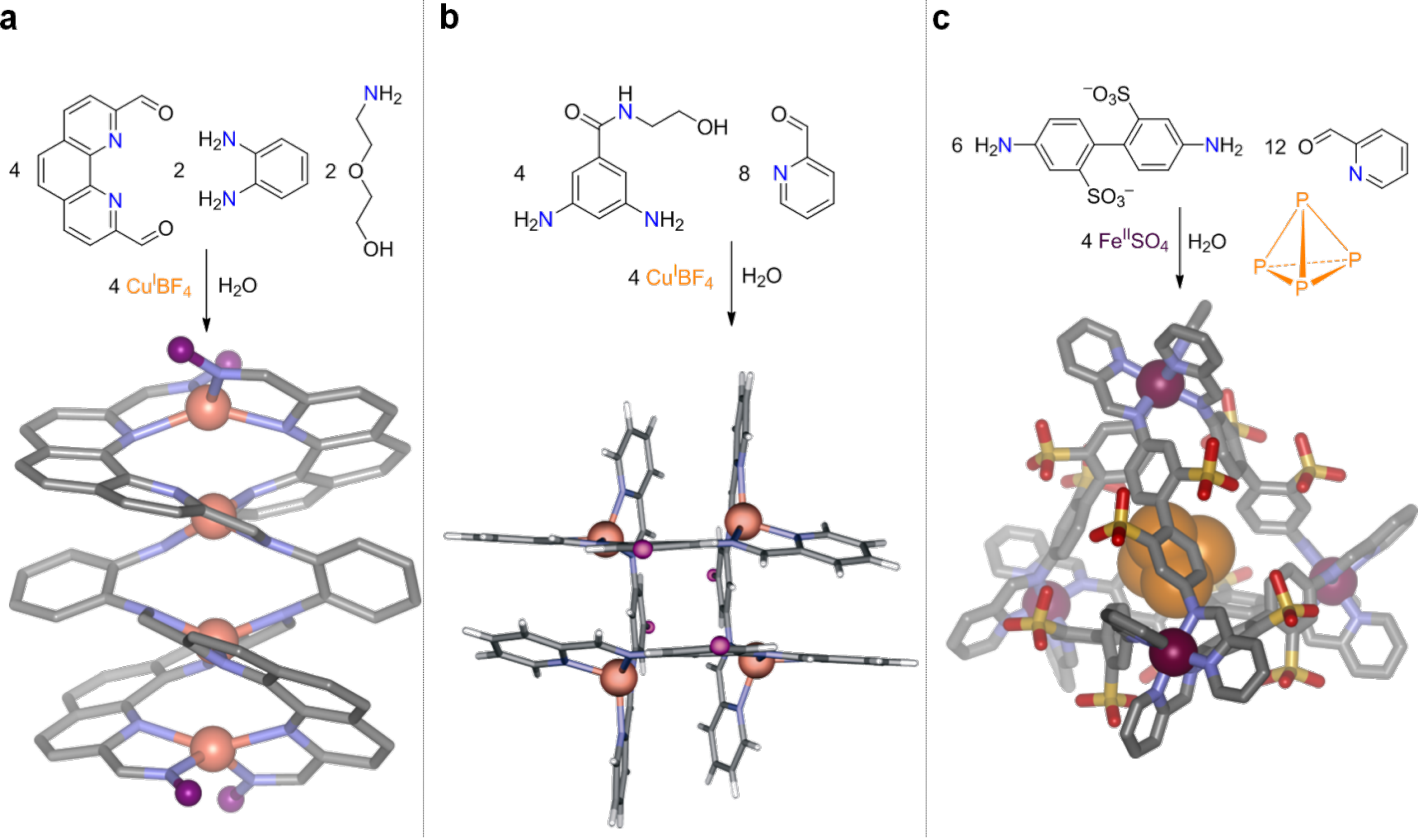
Complex structures prepared from simple subcomponents: a) Cu^I^_4_ helicate [7]; b) Cu^I^_4_ grid [8]; c) Fe^II^_4_ tetrahedral cage [9–10].

This proposal, and the research programme that followed, involved the use of old chemistry – Daryle Busch’s metal-templated imine-bond forming reaction [6] – in new ways. We called it subcomponent self-assembly to emphasize the use of simple precursors to build complex products. The preparations and crystal structures of three of these products are shown in Figure 1.

Each of these products was prepared simply by mixing the precursors shown in water, and each represents a different way to arrange four metal ions in space, going from a one-dimensional linear array (Figure 1a) [7] to a two-dimensional grid (Figure 1b) [8] to a three-dimensional tetrahedron (Figure 1c) [9], which turned out to be capable of binding white phosphorus (P_4_) and rendering it air-stable [10]!

The work went well at Geneva – we were publishing in good journals, and I managed to win a Swiss National Science Foundation Assistant Professorship, which would have allowed me to modestly expand the group. My senior colleagues praised my accomplishments, but advised caution: I was not on a tenure track, and no future at Geneva could be guaranteed.

I thus set about looking for new opportunities, and I applied for an open lectureship at Cambridge. I was delighted to get the job, although it meant leaving behind the funding I had just won in Switzerland.

I hadn’t anticipated the psychological gulf between the relatively rosy Swiss funding situation and the harsh headwinds of UK academia – much less research funding to go round meant for much sharper competition! Proposal ideas that had been funded with great scores in Switzerland were mercilessly skewered when submitted to the Engineering and Physical Sciences Research Council (EPSRC), our primary UK science funder. Each failed proposal hurt badly, but I made common cause with others – banding together with Richard Layfield and Paul Lusby to protest our first grants’ rejection to the head of EPSRC, for example. And I slowly got a sense of what a good grant proposal looked like by UK standards, and how to write one. We kept going in lab, seeking to generate new results of the kind that could underpin a successful UK grant proposal.

The story of P_4_ encapsulation [10] garnered substantial positive attention, and an invited *Nature* Q&A piece on systems chemistry [11] probably helped, too. Ultimate success in attracting funding from the European Research Council (ERC) and EPSRC was the sweeter for the many failures that had preceded it. It has also been a delight to see others making use of subcomponent self-assembly to solve new puzzles, citing our development of the technique [12–25].

**Figure 2:**
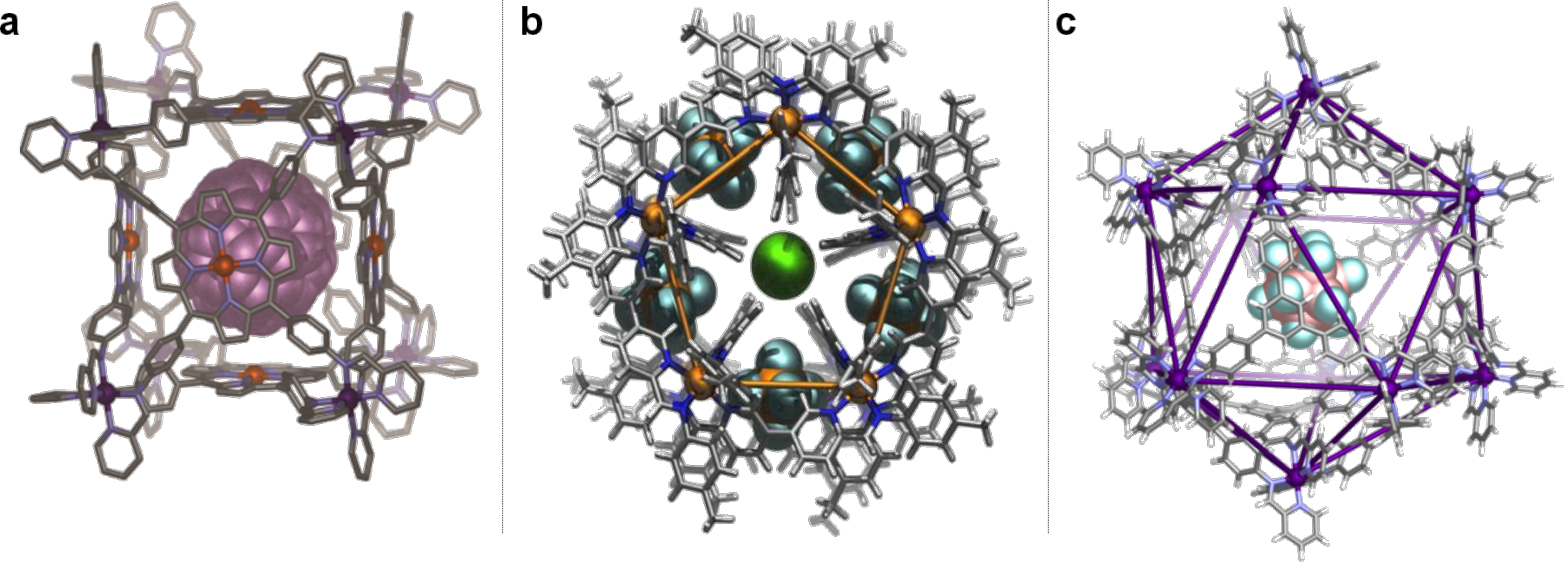
a) A cubic cage [26]; b) a pentagonal prism [27]; c) a *pseudo-*icosahedron [28].

Over the past few years, we have developed a series of new functional structures. A leitmotiv of my group’s work is the integration of a new class of hollow container molecules, invented by my group, into complex and dynamically-responsive systems and materials. Three of these containers are shown below: Figure 2a depicts an Fe^II^_8_L_6_ cubic cage with walls constructed from porphyrins that binds guests such as fullerenes and coronene [26]; Figure 2b shows a Co^II^_10_L_15_ pentagonal prism that embeds five anions, such as PF_6_^−^ (shown) or ClO_4_^−^ in its walls, and a sixth – usually chloride – in its center [27]; Figure 2c illustrates a Fe^II^_12_L_12_ *pseudo*-icosahedron with *mer* stereochemistry to its Fe^II^ centers, shown encapsulating B_12_F_12_^2−^ [28].

We also have a flourishing line of enquiry into conjugated polymers that are held together by metal-ion templation – the structure shown in Figure 1a was a key precursor to this work. In collaboration with Richard Friend in the Physics Department at Cambridge, we have built these polymers into devices that emit white light [29], or that show blue-shifted emission at higher voltages [30], intriguingly.

Key questions that my group and I hope to address over the next few years include:

1. How can we design a system of chemical assemblies to work together in a network, to accomplish a function collectively?
2. Given that increasingly fine-grained control over self-assembled structure is being achieved, how can we design a self-assembling process with a target function in mind, such as light emission or the catalytic transformation of a substrate?

Both of these questions are predicated upon the idea of shifting intellectual effort away from designing and synthesizing complex molecules, and towards understanding and controlling the processes and systems of self-assembly.
